# Primary amputation versus limb salvage in upper limb major trauma: a systematic review

**DOI:** 10.1007/s00590-021-03008-x

**Published:** 2021-05-29

**Authors:** Sandeep Krishan Nayar, Harry M. F. Alcock, Dafydd S. Edwards

**Affiliations:** 1 Centre for Trauma Sciences, Blizard Institute, Queen Mary University of London, 4 Newark Street, London, E1 2AT UK; 2 Department of Trauma and Orthopaedics, Barts Health NHS Trust, London, UK

**Keywords:** Upper limb, Mangled extremity, Amputation, Salvage

## Abstract

**Purpose:**

Severe upper limb injuries can result in devastating consequences to functional and psychological well-being. Primary objectives of this review were to evaluate indications for amputation versus limb salvage in upper limb major trauma and whether any existing scoring systems can aid in decision-making. Secondary objectives were to assess the functional and psychological outcomes from amputation versus limb salvage.

**Methods:**

A systematic review was carried out in accordance with PRISMA guidelines. A search strategy was conducted on the MEDLINE, EMBASE, and Cochrane databases. Quality was assessed using the ROBINS-I tool. The review protocol was registered in PROSPERO.

**Results:**

A total of 15 studies met inclusion criteria, encompassing 6113 patients. 141 underwent primary amputation and 5972 limb salvage. General indications for amputation included at least two of the following: uncontrollable haemodynamic instability; extensive and concurrent soft tissue, bone, vascular and/or nerve injuries; prolonged limb ischaemia; and blunt arterial trauma or crush injury. The Mangled Extremity Severity Score alone does not accurately predict need for amputation, however, the Mangled Extremity Syndrome Index may be a more precise tool. Comparable patient-reported functional and psychological outcomes are seen between the two treatment modalities.

**Conclusions:**

Decision regarding amputation versus limb salvage of the upper limb is multifactorial. Current scoring systems are predominantly based on lower limb trauma, with lack of robust evidence to guide management of the upper extremity. Further high-quality studies are required to validate scoring systems which may aid in decision-making and provide further information on the outcomes from the two treatment options.

## Introduction

The upper limb plays a vital role in our daily function; synergistic movements from the shoulder girdle, elbow, forearm and wrist provide the hand freedom to move around the body during activities of living [1]. Injuries to this vital structure can result in devastating consequences to functional, psychological and social well-being. This is particularly true in cases of a “mangled” upper extremity, defined as injury to three out of four components from assessment of the bones, vessels, nerves and soft tissue [2]. Such injuries often occur in the context of major trauma, defined as an “injury or a combination of injuries that are life-threatening and could be life changing because it may result in long-term disability” [3].

Upper limb injuries of this nature are challenging to manage and deciding which patients would benefit from limb salvage versus amputation is critical [4]. A multidisciplinary approach is required and should take into account the patient’s co-morbidities, pre-injury function and social situation [5]. Salvage surgery is often lengthy and complex, with failure potentially leading to multiple subsequent revision surgeries, which may further exacerbate the negative impact from the trauma. In these cases, primary amputation would be a more suitable path to achieve better functional and psychological recovery.

A number of scoring systems exist to aid in this decision-making, most notably the Mangled Extremity Severity Score (MESS) [6]. However, these scoring systems are based on data from lower limb trauma, therefore extrapolation to upper limb trauma should be undertaken with caution. Furthermore, advances in the management of complex limb injuries over the past 25 years have led to the prognostic value of scoring systems such as the MESS being put into question [7–11].

Guidance on when to carry out limb salvage versus amputation is also available via an algorithm published by the Western Trauma Association in 2012, and more recently guidelines published by the American Academy of Orthopaedic Surgeons in December 2019 [12, 13]. Both documents emphasise that decision-making is complex and often multifactorial. Poor prognostic factors for limb salvage where primary amputation should be considered include severe polytrauma, high-energy blunt mechanisms of injury, shock on presentation, warm ischaemia time greater than six hours and where the limb is attached by only marginal amounts of subcutaneous tissue and/or skin. It should be noted, however, that these guidelines lack high-quality evidence and similarly to the scoring systems are predominantly based on lower limb data.

Regarding outcomes, data from the military on lower limb trauma demonstrates better functional and psychological outcomes following amputation compared to limb salvage [14] whereas similar functional outcomes are seen in the civilian setting [15]. However, given the difference in utilisation, function and demand between the upper and lower limbs, the outcomes from these treatment modalities are likely to differ significantly.

At present, there are no systematic reviews specifically looking at indications for primary amputation versus limb salvage in upper extremity trauma and their associated outcomes. Therefore, a systematic review will aid in providing information on how to approach such situations in an evidence-based manner. The primary aims of this systematic review are to evaluate indications for amputation versus limb salvage in upper limb trauma and whether any existing scoring systems can aid in decision-making. Secondary aims are to assess the functional and psychological outcomes from amputation versus limb salvage.

## Methods

### Protocol and registration

A systematic review of the published literature was carried out in accordance with the Preferred Reporting Items for Systematic Reviews and Meta-Analyses (PRISMA) statement [16, 17]. The review protocol was registered in PROSPERO, the international prospective register of systematic reviews database (registration number CRD42019157078).

### Eligibility criteria

Inclusion criteria were adults (18 years or over) with upper limb trauma at any level from the shoulder to the wrist where primary amputation versus limb salvage was considered. Exclusion criteria were traumatic amputation, limb replantation, hand trauma, children (less than 18 years), reviews, conference abstracts and opinion-based reports.

### Search strategy

A comprehensive search of the published literature on the MEDLINE, EMBASE and Cochrane databases from inception to 1st December 2020 was carried out. The following search terms were used: (upper limb OR upper extremity OR shoulder OR elbow OR arm OR forearm) AND (trauma OR injur* OR mangled) AND (amputat*) AND (salvage OR reconstruct*). The search was performed without date, language, or publication status restriction. An extended search was also conducted on the MEDLINE and EMBASE databases using exploded MeSH and Emtree terms, respectively (“Appendix 1”).

### Study selection

Two reviewers (SN and HA) independently performed eligibility assessment of the articles [18, 19]. This was initially carried out through screening of the article titles and abstracts; the process was completed by full text evaluation. Disagreements between reviewers were resolved via consensus with the senior author. Citation searches were subsequently undertaken to identify any papers not identified from the initial search. Specifically, a backward citation search was carried out to review papers cited by each article, and a forward citation search to review other papers that have cited the included articles.

### Data extraction

A pilot of the data proforma was initially conducted using 5 randomly chosen papers to develop a final proforma. Information collected included: study design, study objective, setting (military versus civilian), sample size, patient demographic information and outcomes.

### Summary measures

Regarding the primary outcome, analysis was carried out in a qualitative manner, evaluating the reasons why one treatment modality was chosen over another, and whether any existing scoring systems were used to aid with decision-making. Considering the secondary outcomes, functional and psychological recovery was assessed based on both objective and subjective measures. From this information, a narrative synthesis of the findings from the included studies was carried out. Result heterogeneity was evaluated to see if quantitative assessment with meta-analysis was possible.

### Risk of bias and quality appraisal

Risk of bias was assessed for each individual study. This was carried out in accordance with the Cochrane Collaboration guidelines using the Risk Of Bias In Non-randomized Studies of Interventions (ROBINS-I) tool. This scores observational studies across seven distinct domains (confounding, participant selection, classification of interventions, deviations from the intended intervention, missing data, measurement of outcomes, and selection of reported results) giving an overall judgement of “low risk”, “moderate risk”, “serious risk” or “critical risk” of bias [20].

## Results

### Study selection

**Fig. 1:**
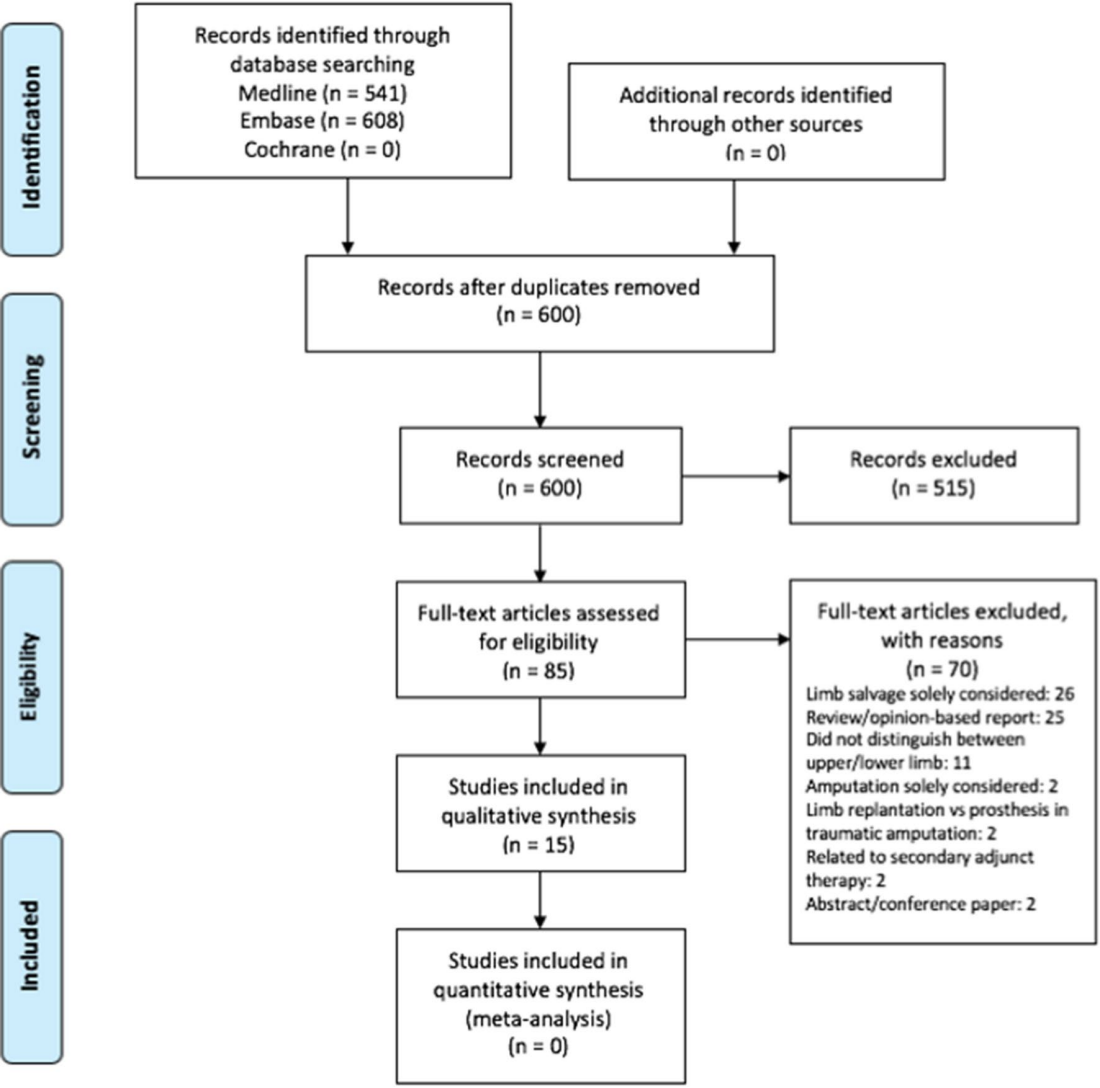
Flow diagram of study selection

A total of 1149 articles were identified from the initial search strategy, of which 549 were duplicates. Following screening of titles and abstracts, 515 papers failed to meet the inclusion criteria. The remaining 85 articles were retrieved for full text review. Of these, 70 were not relevant to the inclusion criteria and were thus excluded. Therefore, a total of 15 papers were included for analysis (Fig. 1). Due to the heterogeneity in outcome measures between studies, a meta-analysis was not feasible.

### Study characteristics

The included studies were published between 2005 and 2019. 14 papers were retrospective cohort studies [21–34] and one was a case series [35]. 13 papers considered patients in the civilian setting [22–29, 31–35], one in the military setting [21] and one looked at both [30]. The number of participants in each study ranged from three to 5260 with a mean of 408 and median of 49 participants. Overall, a total of 6113 participants were included in this systematic review, of whom 141 underwent primary amputation and 5972 underwent limb salvage. The mean age varied from 24.6 to 43.3 years across all studies, with an overall mean of 33.2 years and median of 32.7 years. The majority of cases were in male patients with a mean male percentage of 78%.

14 papers outlined the reason why amputation versus limb salvage was considered [22–35], of which one provided functional outcomes from these treatment modalities [22]. One further paper considered only functional and psychological outcomes following amputation versus limb salvage [21]. The majority of studies did not consider the anatomical level of the trauma, therefore precluding extraction of this data. A summary of all of the study characteristics is shown in Table 1.

**Table 1:** Study characteristics

Authors (year)	Country	Study design	Setting	Mean age (years)	Mean % male	Total sample size	Primary amputation sample size	Limb salvage sample size
Mitchell et al. [21]	USA	Retrospective cohort	Military	30	98%	137	33	104
Kumar et al. [22]	India	Retrospective cohort	Civilian	35.8	80%	10	3	7
Fochtmann et al. [23]	Austria	Retrospective cohort	Civilian	38	72%	54	1	53
Baghi et al. [24]	Iran	Retrospective cohort	Civilian	29	99%	50	1	49
Ege et al. [25]	Turkey	Retrospective cohort	Civilian	24.6	Not provided	30	5	25
Paryavi et al. [26]	USA	Retrospective cohort	Civilian	27.5	Not provided	38	2	36
Franz et al. [27]	USA	Retrospective cohort	Civilian	34	81%	135	1	134
Tan et al. [28]	USA	Retrospective cohort	Civilian	36.9	84%	5260	68	5192
Ball et al. [29]	USA	Retrospective cohort	Civilian	40	47%	18	17	1
Dragas et al. [30]	Serbia	Retrospective cohort	Both	38.7	89%	167	3	164
Ekim et al. [31]	Turkey	Retrospective cohort	Civilian	27.9	88%	49	1	48
Rasouli et al. [32]	Iran	Retrospective cohort	Civilian	27.1	89%	113	3	110
Heis et al. [33]	Jordan	Retrospective cohort	Civilian	32	74%	32	1	31
Joshi et al. [34]	Canada	Retrospective cohort	Civilian	32.7	88%	17	1	16
Togawa et al. [35]	Japan	Case series	Civilian	43.3	25%	3	1	2

### Indications for amputation versus limb salvage

Considering the indications for when primary amputation was carried out, the reason for amputation was provided for 17 individual cases across 11 studies [22–24, 26, 27, 30–35]. In a further three studies, encompassing 90 patients, generalised reasons for amputation were provided [25, 28, 29].

For the individually reported cases: five were due to extensive soft-tissue destruction with signs of ischaemia [23, 30, 31]; four were due to uncontrollable haemodynamic instability [22, 26, 35]; three were due to blunt arterial trauma [32, 34]; two were due to crush injury resulting in a mangled extremity [22, 33]; one was due to an open fracture with severe nerve damage and blood loss [22, 27]; one was due to an open fracture from a blast injury with severe muscle necrosis and shock [22]; and one was due to arterial injury with more than 6 h between injury and arrival to the treating hospital [24].

From the remaining three papers, Tan et al. reported on 5260 patients of whom 68 required primary amputation, stating that blunt arterial trauma was a key indication for amputation [28]. Ball et al. [29] looked at 17 primary amputations, reporting that amputation was carried out in cases where there was concurrent soft tissue, bone, vascular, and nerve (transection) injuries so severe that attempted limb salvage was felt to be potentially harmful. A further paper with five primary amputations reported that amputation was carried out in cases of prolonged hypotension associated with arterial injury [25].

### Use of scoring systems in aiding decision for amputation

Five studies investigated the use of scoring systems to aid in decision-making for amputation versus salvage [22, 23, 25, 29, 35]. All five studies looked at the usefulness of the MESS score, with one study also considering the Mangled Extremity Syndrome Index (MESI) [22]. According to the literature, amputation should be considered in cases where the MESS score is greater than 7 or MESI score greater than 20 [6, 36]. Whilst all five studies found a mean MESS score greater than 7 for patients that underwent amputation, a number of outliers were identified.

Ege et al. reported on 30 patients, five of whom required amputation. They found a mean MESS score of 8.8 for patients that required amputation compared to a score of 5.29 for those that received limb salvage, with one patient requiring amputation despite a MESS score less than 7. They calculated that with regards to determining the need for amputation, the MESS score had a sensitivity of 80%, specificity of 84%, positive predictive value of 55.55% and negative predictive value of 95.45%. Furthermore, they speculated that the most important MESS components associated with amputation were prolonged hypotension and ischaemia from arterial injury [25].

In a study by Fochtmann et al., 17 out of 54 patients had a MESS score greater than 7, including one patient in their study that required a primary amputation. From the remaining 16 patients, nine had successful limb salvage, five died within 72 h due to haemorrhage secondary to the trauma and the remaining two underwent secondary amputation within 2 weeks, one for soft tissue infection and one for vessel occlusion after vessel reconstruction [23]. Another study with 18 patients of whom 17 underwent amputation found a mean MESS score of 9. However, the study did not specify whether there were any cases with a MESS score less than 7 [29]. Furthermore, a case series of three patients demonstrated one patient that underwent an amputation to have a MESS score of 11, and the remaining two patients with MESS scores of 7 and 11 undergoing successful limb salvage [35].

Kumar et al. reported on a total of 10 patients of which three required amputation with MESS scores of 8, 9 and 9, and MESI scores of 22, 21 and 20, respectively. The remaining patients that underwent limb salvage all had a MESS score greater than 7 and MESI score less than 20. Therefore, the MESS score was considered inaccurate for predicting the need for amputation, whereas the MESI score was found to be a more reliable measure [22].

### Functional outcomes from amputation versus limb salvage

Comparison of functional outcomes between the two treatment modalities was addressed in two papers.

The Military Extremity Trauma Amputation/Limb Salvage (METALS) study looked at 135 individuals in the military setting, of whom 33 underwent primary amputation. At 40 months follow up there was no significant difference in self-reported functional outcomes from the Short Musculoskeletal Function Assessment (SMFA) between patients that underwent limb salvage or amputation. However, function was significantly impaired in both groups compared to that of the general population [21].

This finding was replicated in the civilian setting with Kumar et al. similarly showing no difference in self-reported function from the short form-36 (SF-36) between the two treatment groups. Furthermore, in this study, function at 12 months was reported as satisfactory in the majority of patients, with more disability seen in elderly and diabetic patients [22].

### Psychological outcomes from amputation versus limb salvage

Psychological outcomes were solely reported in the METALS study. Their results demonstrated depressive symptoms in 40% of participants, with 12.3% screening positive for possible or probable depression and 19.4% screening positive for PTSD. However, akin to functional outcomes, no statistically significant difference was observed between those that underwent limb salvage or amputation. Key factors related to worse psychological outcomes were increasing age and lack of social support [21].

### Risk of bias

Risk of bias for each study in each individual domain and overall is summarised in Table 2. All of the included studies had moderate to serious risk of bias predominantly due to an inherent risk of confounding, or not controlling for potential confounding factors. Furthermore, all studies were deemed to have moderate risk of bias for selection of participants and classification of interventions. This was because patient selection and the indications for amputation were all considered retrospectively.

**Table 2:** Summary of quality assessment (ROBINS-I)

Authors (year)	Type of bias							Overall risk of bias
	Confounding	Participant selection	Classification of interventions	Deviation from intended intervention	Attrition bias	Detection bias	Reporting bias	
Mitchell et al. [21]	Moderate	Moderate	Moderate	NI	Moderate	Low	Low	Moderate
Kumar et al. [22]	Moderate	Moderate	Moderate	NI	Low	Low	Low	Moderate
Fochtmann et al. [23]	Moderate	Moderate	Moderate	NI	Low	Low	Low	Moderate
Baghi et al. [24]	Serious	Moderate	Moderate	NI	Low	Low	Low	Serious
Ege et al. [25]	Moderate	Moderate	Moderate	NI	Low	Low	Low	Moderate
Paryavi et al. [26]	Moderate	Moderate	Moderate	NI	Low	Low	Low	Moderate
Franz et al. [27]	Serious	Moderate	Moderate	NI	Low	Low	Low	Serious
Tan et al. [28]	Serious	Moderate	Moderate	NI	Low	Low	Low	Serious
Ball et al. [29]	Serious	Moderate	Moderate	NI	Low	Low	Low	Serious
Dragas et al. [30]	Moderate	Moderate	Moderate	NI	Low	Low	Low	Moderate
Ekim et al. [31]	Serious	Moderate	Moderate	NI	Low	Low	Low	Serious
Rasouli et al. [32]	Serious	Moderate	Moderate	NI	Low	Low	Low	Serious
Heis et al. [33]	Serious	Moderate	Moderate	NI	Low	Low	Low	Serious
Joshi et al. [34]	Serious	Moderate	Moderate	NI	Low	Low	Low	Serious
Togawa et al. [35]	Moderate	Moderate	Moderate	NI	Low	Low	Low	Moderate

Risk of bias due to missing data (attrition bias) was considered low in all cases due to the presence of complete data sets, with the exception of one study in which there was a participation rate of 59.8%. However, in this study further analysis of the cohort did not find any major differences between the patients who participated in the study and those who did not consent or could not be located, thus precluding it from being at risk of serious bias [21].

Risk of bias in the measurement of outcomes (detection bias) was deemed low for all studies as the methods of outcome assessment were comparable across intervention groups and outcome measures were unlikely to be influenced by knowledge of which intervention was carried out. Similarly, risk of bias in selection of the reported results (reporting bias) was deemed low for all studies as no gaps in reported outcomes were identified.

## Discussion

This review has highlighted that the indications for amputation are varied, complex and multifactorial. Key factors to consider can be broadly categorised into three subgroups: global patient factors, limb-specific factors and mechanism of injury factors. The main global patient factor is the presence of uncontrollable haemodynamic instability. Limb-specific factors include extensive and concurrent soft tissue, bone, vascular and/or nerve injuries, and prolonged limb ischaemia. Mechanism of injury factors are blunt arterial trauma and crush injuries. Amputation was generally undertaken where there were elements from at least two of these subgroups present.

Regarding the use of scoring systems, evidence from the literature suggests that the MESS scoring system alone is neither specific nor sensitive enough to predict the need for primary amputation. In contrast, the MESI score appeared to be a more accurate tool. However, this is based on a single retrospective study and further studies including a large prospective study are required in order to validate this finding. Another tool which has been recently developed specifically for upper limb trauma is the Mangled Upper Extremity Score (MUES) [37]. This was created from retrospective evaluation of 76 patients over a 10-year period. The score assigns one point each for: age greater than 40 years, need for fasciotomy, need for bony fixation, presence of a bony defect, need for revascularisation, crush injury mechanism, presence of a degloving or avulsion injury, and a soft tissue defect greater than 50 cm^2^, with the authors demonstrating a score of 6 or more to be an accurate indicator for when to carry out amputation. However, akin to the MESI score, this tool is based on a single retrospective study, thus further studies are required to ensure its validity.

Considering the secondary outcomes, very few studies have directly compared the long-term functional and psychological outcomes from amputation versus limb salvage in the upper limb. From the two studies included in this systematic review, the outcomes from the two treatment modalities are comparable. This contradicts the current practice favouring limb salvage over amputation, with previous suggestion in the literature that a “bad hand” may be more functional than a “good amputation” [21, 38]. Furthermore, these results contrast findings from a systematic review by Otto et al. evaluating outcomes from replantation versus prosthesis fitting following traumatic arm amputation, in which better outcomes were seen from replantation [39]. A large study evaluating long-term outcomes from upper limb amputation versus limb salvage in the civilian setting, analogous to the Lower Extremity Assessment Project (LEAP) study for lower limb trauma, is warranted [15].

In general, a much higher proportion of patients with a mangled upper extremity undergo limb salvage when compared to the mangled lower extremity. Whilst one reason for this is the previously mentioned belief that the limb should aim to be preserved whenever possible unless in extremis, another important factor is that limb salvage for the mangled upper extremity tends to be more successful with less complications when compared to the lower limb [15, 37, 40, 41]. This is due to anatomical differences with a greater collateral circulation in the upper limb, particularly from the rich collateral circulation of the brachial artery, allowing for more revascularisation options and a greater time until critical limb ischaemia develops [2, 22, 42, 43].

In recent years there has been significant innovation and development of surgical techniques for limb salvage and prosthesis technology. This includes the use of targeted muscle reinnervation, resulting in amplification of nerve motor signals in the amputated limb allowing for more intuitive control of prosthetic arms [44]. Furthermore, this can be coupled with enhanced myoelectric prostheses, resulting in even greater dexterity and functionality, as well as the use of osseointegration devices to prevent socket fit issues and further enhance function [45, 46]. The use of this technology may result in a lower threshold to carry out amputation in cases where limb salvage is controversial and to prevent the sequalae of multiple revision surgeries.

The recommendations that emerge from this systematic review of the currently available literature are summarised in Table 3.

**Table 3:** Author recommendations

Primary amputation in upper limb major trauma—decision making variables:
1. Global patient factors:
Uncontrollable/refractory haemodynamic instability
2. Limb-specific factors:
Extensive injury to at least three out of four components from:
(1) Soft tissue
(2) Bone
(3) Vessels
(4)Nerves
Prolonged limb ischaemia
3. Mechanism of injury factors:
Blunt arterial trauma
Crush injury
Amputation should be considered in cases where there are elements from 2 or more of the above subgroups (level IV evidence)
Use of scoring systems:
Absolute scores should not be used to decide on the need for amputation (level IV evidence)
MESS score < 7 and/or MESI score < 20 may suggest limb salvage to be a plausible option (level IV evidence)
Other considerations:
Decision on when to amputation should be carried out on a case by case basis
Decision making should involve a multidisciplinary team (including consultant orthopaedic/vascular/plastic surgeons and those involved in aftercare/rehabilitation)
Patient choice should be taken into consideration and respected where possible for individuals with capacity to make an informed decision

This systematic review is limited by the lack of available high-quality studies in the literature. All included articles are graded as level IV evidence, undermining the quality of the overall results. Furthermore, due to heterogeneity in outcome measures between each study, interpretation bias might have occurred in this review. In order to minimise this risk, a standardised data extraction form was used. Additionally, the review did not consider the different anatomical levels of amputation. This relates to a large proportion of the included studies not referencing this information and therefore precluding extraction of this data.

These limitations highlight the need for large high-quality studies to further enhance our understanding of when to carry out amputation versus limb salvage. This is particularly important with the emergence of technological advances in upper limb prosthetics.

## Conclusion

Whilst limb injuries are a common feature in trauma, major upper extremity trauma where there is a decision on whether to carry out amputation versus limb salvage is a rare occurrence [23, 37], which is reflected by the lack of high-quality evidence available in the literature. This systematic review has demonstrated that there are a number of factors to consider when making this decision. It is therefore fundamental that there is collaboration and consensus between the orthopaedic, vascular and plastic surgeons, as well as input from the wider multidisciplinary team involved in patient aftercare and rehabilitation. Current scoring systems do not provide accurate prognostic information, and thus must be interpreted in the context of the wider clinical picture. Furthermore, this systematic review has demonstrated comparable functional outcomes between the two treatment options, however, the individual patient must be taken into account in each case.

There is a clear need for further high-quality studies to enhance our understanding of when to carry out primary amputation in order to achieve optimal outcomes, as well as to validate any scoring systems which may aid in decision-making. Finally, with recent developments in what is technologically and surgically possible, this field is likely to advance greatly over the coming years, further emphasising the importance and need for continued research.

## Appendix 1: Full search strategy

Initial search on the MEDLINE, EMBASE and Cochrane databases:

(upper limb OR upper extremity OR shoulder OR elbow OR arm OR forearm) AND (trauma OR injur* OR mangled) AND (amputat*) AND (salvage OR reconstruct*).

Extended search on the MEDLINE database with exploded MeSH terms:

(*AMPUTATION/ OR "AMPUTATION, TRAUMATIC"/) AND ("UPPER EXTREMITY"/ OR *ARM/ OR *ELBOW/ OR *FOREARM/ OR *SHOULDER/) AND "LIMB SALVAGE"/).

Extended search on the EMBASE database with exploded Emtree terms:

(*AMPUTATION/ OR *"ARM AMPUTATION"/) AND (*"UPPER LIMB"/) AND (*"LIMB SALVAGE"/).
